# On the Generalizability of Time-of-Flight Convolutional Neural Networks for Noninvasive Acoustic Measurements

**DOI:** 10.3390/s24113580

**Published:** 2024-06-01

**Authors:** Abhishek Saini, John James Greenhall, Eric Sean Davis, Cristian Pantea

**Affiliations:** Los Alamos National Laboratory, Los Alamos, NM 87544, USAesdavis@lanl.gov (E.S.D.);

**Keywords:** time-of-flight, convolutional neural networks, CNN-ToF, generalizability

## Abstract

Bulk wave acoustic time-of-flight (ToF) measurements in pipes and closed containers can be hindered by guided waves with similar arrival times propagating in the container wall, especially when a low excitation frequency is used to mitigate sound attenuation from the material. Convolutional neural networks (CNNs) have emerged as a new paradigm for obtaining accurate ToF in non-destructive evaluation (NDE) and have been demonstrated for such complicated conditions. However, the generalizability of ToF-CNNs has not been investigated. In this work, we analyze the generalizability of the ToF-CNN for broader applications, given limited training data. We first investigate the CNN performance with respect to training dataset size and different training data and test data parameters (container dimensions and material properties). Furthermore, we perform a series of tests to understand the distribution of data parameters that need to be incorporated in training for enhanced model generalizability. This is investigated by training the model on a set of small- and large-container datasets regardless of the test data. We observe that the quantity of data partitioned for training must be of a good representation of the entire sets and sufficient to span through the input space. The result of the network also shows that the learning model with the training data on small containers delivers a sufficiently stable result on different feature interactions compared to the learning model with the training data on large containers. To check the robustness of the model, we tested the trained model to predict the ToF of different sound speed mediums, which shows excellent accuracy. Furthermore, to mimic real experimental scenarios, data are augmented by adding noise. We envision that the proposed approach will extend the applications of CNNs for ToF prediction in a broader range.

## 1. Introduction

Acoustic time-of-flight (ToF) prediction is an essential tool in non-destructive evaluation (NDE) techniques for a wide range of applications, including noninvasive measurement for in situ process control [1,2,3], biohazard monitoring [4], chemical reaction monitoring [5], structure monitoring [6,7,8] and identification or classification of materials inside sealed containers [9,10]. The ToF refers to the time required by acoustic waves to travel between source and receiver through a given medium. Typically, the ToF-based techniques are the first choice for inspection and characterization because they are sensitive to any change in acoustic speed (i.e., to material), they are relatively simple to interpret, and they enable rapid measurements. One popular ToF technique is travel time tomography, which reconstructs the sound speed profile of the medium [11] that can then be used for correlating physical properties of interest, such as temperature, material phase, species concentration, etc. In many NDE applications, the materials are enclosed in containers (in this work “containers” refer to cylindrical shape containers), and the material is attenuative.

In closed containers, “bulk waves” propagating through the enclosed material can interfere with the guided waves propagating around the container wall. These guided waves inhibit identifying and measuring the ToF of the bulk waves. When using a broad-band (high frequency and short burst duration) signal, time-gating is used to identify bulk and guided waves. However, in attenuating materials, lower excitation frequencies must be used to mitigate attenuation. This results in longer burst durations and, thus, more overlap between bulk and guided wave arrivals. Several other techniques were proposed for detecting the bulk waves through cylindrical containers in the presence of guided waves [1]. Baseline subtraction was used to separate the bulk and guided waves by first measuring the guided waves from an empty container while keeping a thin fluid residue on the container wall and then subtracting the baseline signal from the combined wave signal (bulk and guided) obtained from a fluid-filled container [1]. However, this is not feasible in many applications where operators do not have access to the empty container to collect baseline measurements. Alternatively, a broadband linear chirp excitation technique was implemented to exploit the dispersive behavior of the guided waves [9]. The frequency-dependent characteristic of the guided waves distorts and disperses the guided waves, whereas the bulk waves remain undistorted. The undistorted bulk waves were then identified using cross-correlation. However, this typically requires high frequencies and bandwidths (>1 MHz), which makes it infeasible for attenuating materials or large containers. In recent years, data-driven techniques, such as machine learning (ML)-based models, especially convolutional neural networks (CNNs), have shown promising results in acoustic imaging and signal processing [12,13,14,15,16,17,18,19,20,21,22]. CNNs are distinguished from other neural networks by their superior performance with image, speech, or audio signal inputs. CNNs can solve complex problems by learning the descriptive functional behavior between input and output, which can be challenging to solve using conventional techniques. They carry this out by extracting features through multiple layers of convolution, nonlinear activation, and pooling, followed by linear classification or regression [23].

A ToF-CNN was proposed for measuring bulk wave ToF through the material enclosed in cylindrical containers and validated for experimental data [10]. The problem of estimating ToF was split into two steps. In the first step, a small time-windowed segment of the acoustic measurement was passed to the CNN, which would determine the likelihood that the time window contained a bulk wave arrival. Next, after evaluating each windowed region of an acoustic measurement, the ToF was measured as the time for which the arrival likelihood was highest. Splitting the ToF measurement into two steps provides three main advantages: (1) it reduces the CNN complexity by reframing a regression problem (predicting ToF) as a binary classification problem (predicting whether a time window contains a bulk wave arrival), (2) using small time windows removes bias from the dimensions and materials of the training data, and (3) dividing each acoustic measurement into small time windows increases the amount of training data. However, the demonstration of the ToF-CNN was limited to nominally identical containers. In practice, it is likely that an operator will encounter containers that differ from those used to train the ToF-CNN, in terms of container size and internal material. Thus, there is a need to analyze the generalizability of the ToF-CNN method with respect to training/testing set parameters such as container dimensions and internal material properties for a broader application. In general, the performance of ML models degrades if (i) the dataset size is not large enough (overfitting), (ii) the features in the dataset do not contain enough information to explain the outcome, and (iii) the ML model is not sufficiently complex to represent the relationship between the features and the outcome (underfitting) [24,25,26,27,28,29]. Collecting sufficient labeled data to overcome these issues in NDE applications presents several challenges due to unknown properties inside the structure, a lack of realistic training cases due to rare conditions, as well as challenges in obtaining real samples for experiments. Furthermore, in high-dimensional data space, the inadequate training samples may span in a small subspace and provide weak constraints on the possible decision boundaries learned by the network, which can lead to poor performance [30,31,32]. Therefore, it is essential to understand the influence of training data on ML model performance for scientific as well as practical benefits.

In the field, an NDE method is useful if it can work on structures with a broad range of parameters that the method likely had not encountered during training. The ability to perform well on new data is known as “generalizability”. In most generalizability studies, the dataset is fixed, and model hyperparameters are tuned to maximize the generalizability on some randomly selected subset of the training data. For the case of developing the ToF-CNN as an NDE technique, the size of the training dataset is not fixed, i.e., we can always collect additional measurements, but we need a method to determine how many data samples are sufficient. Additionally, traditional generalizability studies assume that test samples are drawn from the same distribution as the training samples. In practice, it is challenging to know what the distribution of dimensions and material properties will be encountered in future NDE measurements. Thus, we need a way to determine the range of parameters over which the CNN is expected to perform with a specified level of accuracy. We investigate the model performance by training the CNN on a fraction of data with the smallest diameters, and then testing the CNN using the remaining data samples. We then repeat this process, training on data from the largest containers. This exercise enables quantifying how the expected error level is affected by the amount of parameter separation between the training and testing datasets. In practical terms, it enables an operator to know the range of container sizes that could be measured within a given level of expected accuracy.

Thus, the novel contributions of this work are to (1) develop a methodology to assess how generalizability is affected by training dataset size and differences in training/testing dataset parameter distribution, and (2) quantify the ToF-CNN accuracy and robustness to demonstrate its potential for a broad range of NDE applications. The remainder of the manuscript is organized as follows: Section 2 presents the methodology, including data generation, data preprocessing and augmentation. Results and discussions are illustrated in Section 3, followed by conclusions in Section 4.

## 2. Methodology

To assess the generalizability of the ToF-CNN, we measure its performance in situations where the training and testing containers are not identical and even follow different distributions, i.e., for different container diameters, thicknesses, and internal material sound speeds. The model performance was evaluated by adjusting the training dataset size and distribution and measuring the resulting change in test accuracy. We used finite element simulations to generate synthetic datasets that closely match experimental data. Additionally, data augmentation is incorporated for generating further labeled datasets by adding noise to the data to expand database diversity and better represent experimental data. Data augmentation enlarges the number of training samples, which can improve generalization.

### 2.1. Simulated Data Using Finite Element Method

**Figure 1:**
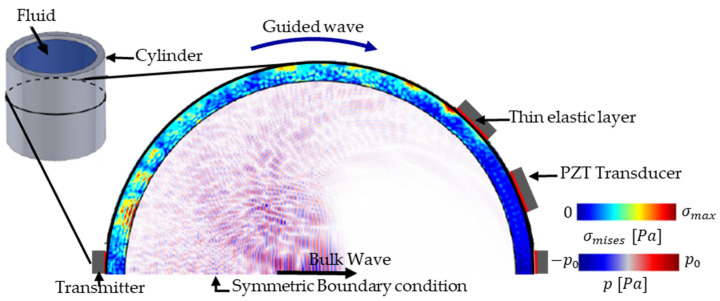
Schematic of the symmetric FEM model of the cylindrical container. A wavefield snapshot at an arbitrary time shows the bulk and guided waves.

We simulate the acoustic bursts through a 2D cross-section of the cylindrical container using the finite element method (FEM) software COMSOL 6.1. A fluid-filled axisymmetric aluminum 6063 containers with varying dimensions (inner diameter and thickness) is modeled, as displayed in Figure 1. A symmetric boundary condition is applied, and the half-space is modeled to reduce computational complexity. We attached five receivers opposing one transmitter (Lead Zirconate Titanate, PZT-5J) with a cross-section of 7 mm diameter and 0.5 mm thickness to the outer circumference of the container. We note that, for the half-space simulation, we only simulate three of the receivers (upper receivers in Figure 1), where the remaining two receivers (lower receivers not shown in Figure 1) are assumed to be symmetrical. We note that, for the case of a homogeneous fluid in the container, one receiver is theoretically sufficient for accurately measuring sound speed. We note that, for the case of a homogeneous fluid in the container, one receiver is theoretically sufficient for accurately measuring sound speed. We include multiple receivers for three reasons: (1) to ensure that the ToF-CNN technique can be applied to materials that are not homogeneous and require multiple receivers, (2) to add robustness to the ToF-CNN technique to account for faults in any one receiver, and (3) because including data from three receivers triples the dataset size and the variability in the bulk and guided wave arrival times and amplitudes, which leads to improved model generalizability.

We excite the transmitter with a Gaussian-modulated cosine voltage $v_{ex}\left(t\right)$ defined as

(1) $$v_{ex}\left(t\right)=v_{0}e^{\left(-\frac{{\left(t-T/2\right)}^{2}}{2\sigma_{ex}^{2}}\right)}\cdot cos\left(2\;\pi f_{c}\left(t-T/2\right)\right),$$

where $v_{o}$ is the amplitude,$\;f_{c}$ = 350 kHz is the center frequency, $\sigma_{ex}=150\;\mathrm{kHz}$ is the standard deviation, *T* is the burst duration, and t is time. Acoustic waveforms are obtained by solving time-domain coupled acoustic-elastic wave propagation in a closed container. Triangular elements are used in the mesh with the size of at least 15 elements per wavelength of the smallest wavelength at a given center frequency to reduce numerical error. Besides the spatial, the temporal discretization also satisfied a sampling criterion $\Delta t\leq {\left(10(f_{c}+\sigma_{ex}\right))}^{-1}$ to ensure the stability of the finite element method.

In experiments, the largest deviations are often caused by the transducer coupling, which can change the ringing in the transducer. To replicate realistic transducer attachment via adhesive, we model the transducer-container coupling as a thin elastic layer (TEL), where the layer thickness is varied to represent variability in experimental attachment. TEL represents the elastic and damping conditions between two boundaries. In an interior boundary, the TEL separates the displacements between two sides of the boundary. The two boundaries are then linked by elastic and viscous forces of equal magnitude but opposite directions, which are proportional to the relative displacements and velocities. We defined the TEL material properties for random thickness ($\Gamma_{TEL}$), Young’s modulus (E=3 GPa), Poisson’s ratio (ν=0.36), and the thickness of the TEL to resemble an epoxy adhesive. From these material properties, we compute the spring constant per unit area in directions normal ($k_{n}$) and tangential ($k_{t}$) of the TEL as: $k_{n}=\frac{E\left(1-\nu \right)}{\Gamma_{TEL}\left(1+\nu \right)\left(1-2\nu \right)}$; $k_{t}=\frac{E}{2\Gamma_{TEL}\left(1+\nu \right)}$. The values of $k_{n}$ and $k_{t}$ stiffness per unit area are used for spring constant per unit area in the TEL boundary condition. A multiphysics approach was used to couple acoustic-structure boundary and piezoelectric effect in simulations. For each wave propagation simulation, we randomly sampled the outer diameter, thickness of container wall, TEL thickness, and internal material sound speed from normal distributions with mean and standard deviation listed in Table 1, based on common values in NDE applications. We simulated 1089 bursts for a sound velocity of 1498 m/s, each of which returned 3 waveforms (one per receiver), where each waveform was then divided into 952 windows with an overlap of 99-time samples. This resulted in a total of 3,110,184 data samples (one windowed waveform $X_{q}$ plus true arrival $y_{q}$ per sample).

**Figure 2:**
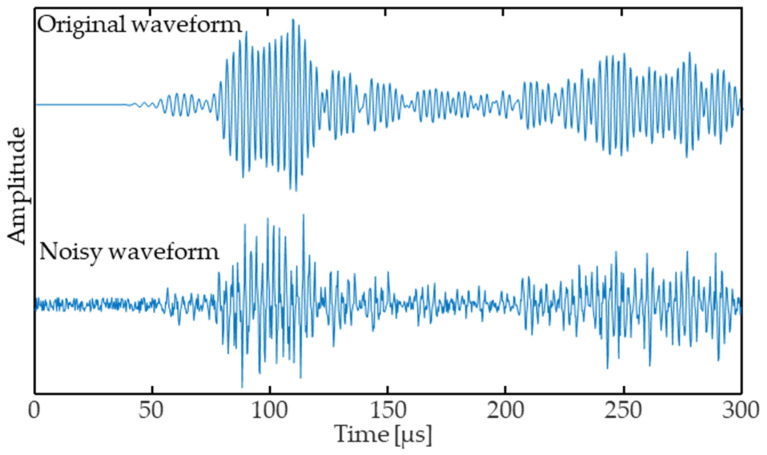
Example original waveform and waveform with noise.

Additionally, experimental data often contains noise due to, e.g., ambient vibrations and electrical noise in the wires and electronics. To improve the generalization ability of the ToF-CNN, we add normally distributed random noise, and we empirically set the noise amplitude to be 4.5% of the average value of the maximum amplitude (3.33 × 10^4^) of the complete dataset to approximate noise levels found in experiment measurements. Raw signals are normalized with the maximum amplitude for further data processing, as discussed in Section 2.2. Figure 2 shows the original waveform and the noisy waveform.

### 2.2. Data Preprocessing

For each of the simulated waveforms, we approximate the “true” ToF as

(2) $$ToF_{true}=\frac{d_{rt}}{c_{f}}+2\Gamma \left(\frac{1}{c_{m}}-\frac{1}{c_{f}}\right),$$

where $d_{rt}$ is the distance from the transmitter to the receiver, Γ is the thickness of the container wall, $c_{f}$ and $c_{m}$ are the sound speed in fluid and the longitudinal sound speed of the container material, respectively. To measure the arrival times, we utilize a CNN to predict the likelihood of a burst arrival $P_{arr}$ at time *t*. We define the true burst arrival likelihood as

(3) $$P_{arr}=exp\left(-\frac{{\left(t-ToF_{true}\right)}^{2}}{2\sigma_{ToF}^{2}}\;\right),$$

We simplify the problem of predicting $P_{arr}$ (a regression problem) to a binary classification problem. In this problem, we approximate the bulk wave arrival likelihood as a binary label

(4) $$y\left(t\right)=\left\{\begin{array}{c} 1{\;\mathrm{if}\;P}_{\mathrm{arr}}\left(t\right)\geq 0.5\\ \;0{\;\mathrm{if}\;P}_{\mathrm{arr}}\left(t\right)<0.5 \end{array}\right.,$$

which indicates if the probability of a bulk wave arrival at time *t* is greater than 50%.

**Figure 3:**
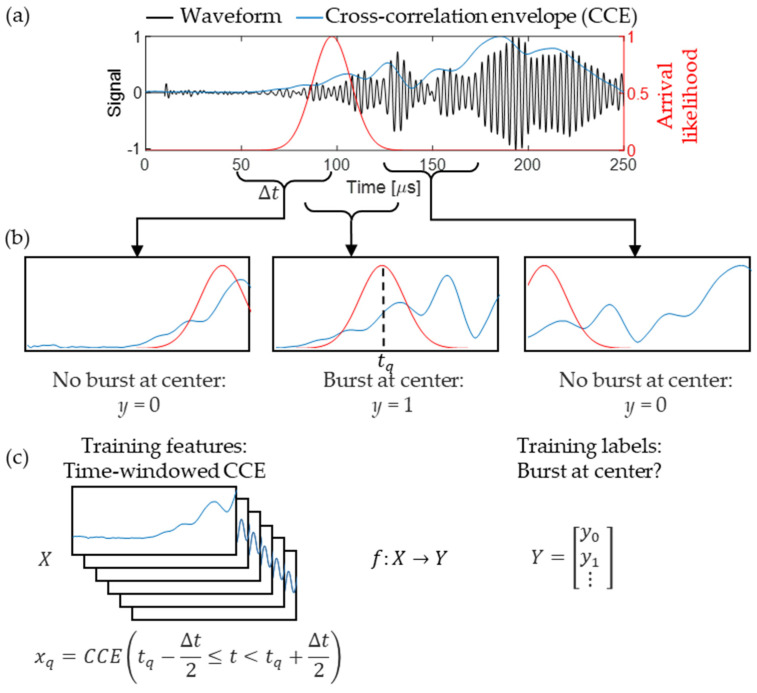
Acoustic data preprocessing for CNN learning. (**a**) Measured acoustic waveform and the corresponding cross-correlation envelope and burst arrival likelihood. (**b**) Example of the time-windowed data with labels indicating whether there is a burst arrival at the time window center. (**c**) Illustration of CNN inputs *X* and outputs *Y* created from a single acoustic waveform.

Figure 3a shows an example of the received waveform (black) with the bulk wave burst arrival likelihood (red). We then compute the cross-correlation envelope (CCE) between the received waveform $v\left(t\right)$ and the excitation $v_{ex}\left(t\right)$, defined as

(5) $$CCE\left(t\right)=\left|H\left(v\left(t\right)⋆v_{ex}\left(t\right)\right)\right|,$$

where $H\left(\cdot \right)$ indicates the Hilbert transform, and ⋆ indicates cross-correlation. The CCE is used as input data instead of the raw acoustic waveform because CCE improves the signal quality by excluding the content outside of the excitation bandwidth while preserving the amplitude information. This procedure also gives the flexibility to downsample longer data without losing useful information, which leads to improved CNN generalizability. It should be noted that the CCE signal may result in lower ToF precision.

We further simplify the sequence-to-sequence problem of detecting the bulk wave arrival *y* at all times *t* to a traditional singular classification problem, wherein we observe small time windows and classify whether they contain a bulk arrival. To achieve this, we split the CCE signal into time-windowed segments with span Δt, centered at $t_{q}$ for the *q*th window, i.e., $x_{q}=CCE\left(t_{q}-\frac{\Delta t}{2}\leq t<t_{q}+\frac{\Delta t}{2}\right)$. We then define a scalar label ($y_{q}$) for each window *q* indicating whether there is a bulk wave arrival at the center of the time window (Figure 3b).

Here, the window span Δt and the amount of overlap (overlap of 99 samples was used in this study) between windows are treated as hyperparameters. Time-windowing assists in reducing any arrival time biases while training because the CNN does not receive any information about what time *t_q_* in a given time window is centered around. Figure 3c shows a single CCE divided into multiple inputs X and scalar outputs Y.

### 2.3. Convolutional Neural Network Structure

**Figure 4:**
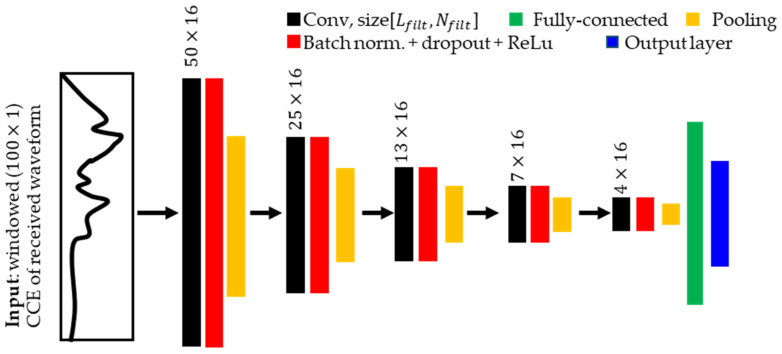
Schematic representation of the CNN used in bulk wave arrival prediction.

Figure 4 shows a schematic of a CNN architecture containing a series of layers including, convolution (black), batch + dropout + ReLu (red), pooling (yellow), fully connected (green), and output layers (blue).

The first element of the CNN is convolutional (Conv) layers that convolve an input signal with a given number of convolution filters to produce filtered signals that are then passed to the next layer through batch normalization (or norm), dropout and activation functions. Batch norm standardizes the values of hidden layer outputs for each batch with its mean and standard deviation. Dropout is used to randomly drop a predefined ratio of connections in a neural network to prevent overdependence on any one neuron. An activation function introduces nonlinear scaling that has been shown to improve CNN performance [33]. We use the rectified linear unit (ReLU) activation function, which helps to create sparse representations of the filtered signals to improve learnability [34].

The second element of our CNN is pooling layers, introduced between two conv layers. Max pooling is used to pick the maximum value from the patch of the input data and summarize these values into a feature map, which maintains the most significant features of the input while reducing its dimensions. The pooling layer also removes sensitivity to small translations of the input signals. Thus, the pooled features are smaller and less sensitive to input signal distortions while preserving critical information from the original signal [29]. Additionally, pooling allows CNN to be more robust to learn invariant features and avoid overfitting, meaning the network will be able to extract features from the signal of interest regardless of the feature position in the signal [35].

The third element of the network is a fully connected dense layer, incorporated into the last layer of the convolutional neural network. A sigmoid activation function is applied in the last layer to scale the output labels between 0 and 1.

Our CNN utilizes 5 Conv layers, each of them with 16 1D convolution kernels ($N_{filt}$) of length ($L_{filt}$) 50. We minimize binary cross-entropy loss by iteratively updating the learnable parameters such as weights and biases in each layer using an adaptive moment estimation, or Adam optimizer over 15 epochs (iteration steps) for each training run. Model checkpointing is used to monitor the model performance after every epoch, and the best model is saved for each dataset size.

To predict ToF, for a test waveform, we break up the waveform CCE into overlapping time-windowed samples (99-time samples), centered at times $0\leq t_{q}\leq t_{f}$, and then use the CNN to predict the likelihood of a bulk arrival *Y*(*t_q_*) at each time *t_q_*. Finally, the bulk wave ToF is estimated by computing the time *t_q_* with the highest likelihood $Y\left(t_{q}\right)$, given by.

(6) $$ToF=\underset{t_{q}}{\mathrm{argmax}}\;Y\left(t_{q}\right)$$

**Figure 5:**
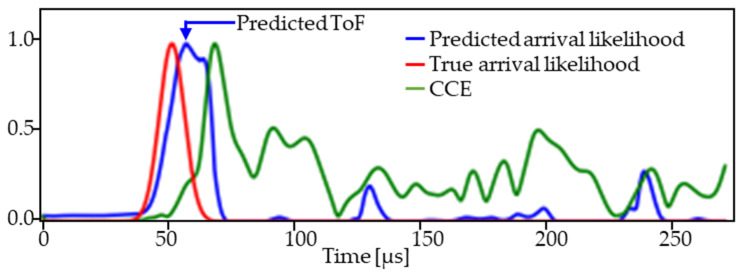
Superimposed comparison of test waveform (CCE) with true and predicted arrival likelihood.

Figure 5 shows the predicted (blue) and true ToF (red) for an example test CCE (green) for a transmitter–receiver pair. We observed good agreement between the predicted and true ToFs.

## 3. Results and Discussion

In the field, operators will likely encounter containers with parameters (dimensions or material properties) that are not found in the training dataset. Intuitively, if the training dataset is too small, the model will overfit, resulting in low prediction accuracy. Similarly, we expect low accuracy if the field container is not drawn from the same distribution of parameters as the training data, e.g., it is significantly larger or smaller than the training data containers. We analyze the ToF-CNN performance in these situations by investigating the influence of training dataset size, and parameter distribution separation between training and testing datasets. The goal is to examine the impact of dataset size and dataset type (data distribution) on the prediction performance. The network performance is evaluated with respect to root mean square error (RMSE).

### 3.1. Influence of Dataset Size

**Figure 6:**
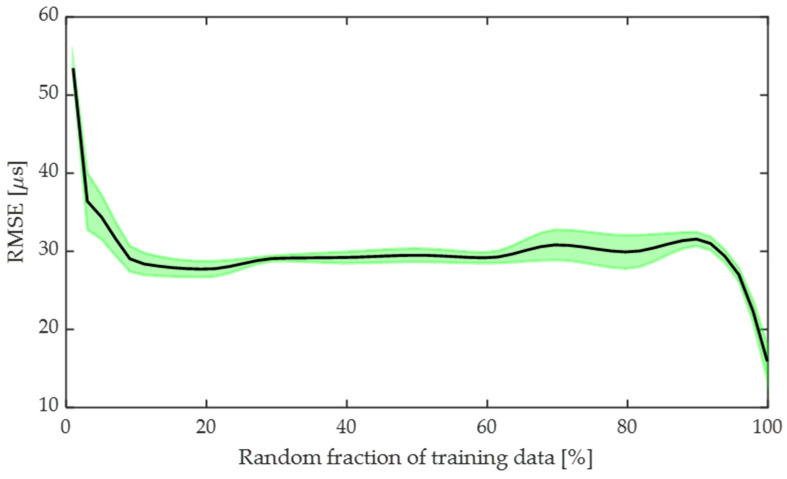
Influence of dataset size on testing accuracy. Plot illustrates the mean (black line) with 95% confidence interval (green shaded area) RMSE of the ToF.

To investigate the impact of dataset size on model accuracy, the ToF-CNN model was trained using 1% to 99.9% of the total dataset, sampled randomly, and the remaining dataset was used for testing. The total dataset contains the acoustic burst (CCE) for all the diameters as mentioned in Section 2.1 and Table 1 using a single medium velocity of 1498 m/s. Figure 6 shows the mean (line) with 95% confidence interval (shaded area) RMSE of the ToF, obtained by investigating the training and testing 10 times. We observe that the RMSE begins to converge after 10% of training data and then dramatically reduces when training on >90% of the total dataset. This indicates that the ToF-CNN is able to represent the distribution of waveform time windows once it has “seen” 90% of those samples during training. It may also be possible that the model “memorizes” rather than “learns” the feature when training on a very large dataset and testing on a small dataset. Additionally, we observe an increase in the test RMSE when using around 70% to 80% of the training data. This is likely due to the fact that 81.5% of the data windows are quite similar, and approximately 18.5% of the datasets are outlier data points, which are relatively similar to each other but dissimilar to the majority of the dataset. Once enough (>10%) data are included in the training dataset, the CNN is no longer overfitting, and the training set contains similar data points to the majority of the test data points. But once the training dataset fraction increases to 70–80%, then test sets are much smaller, so any outlier test samples will have more weight when calculating the RMSE. The presence of outliers can be observed from the wide band of errors, seen in between 70 and 80% of the dataset. Finally, once the fraction of training samples is >90%, whenever there is an outlier sample in the test set, it is highly likely that the CNN will have encountered another similar outlier sample during training.

### 3.2. Influence of Training-Testing Dataset Distribution Separation

**Figure 7:**
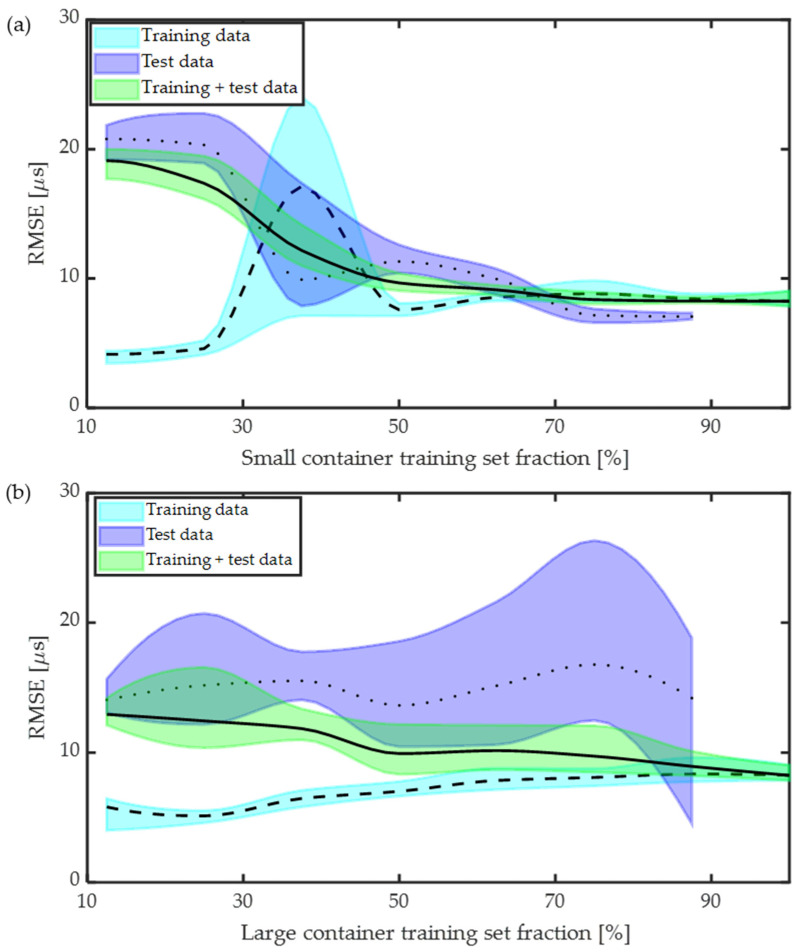
Performance evaluation of CNN for different dataset sizes: (**a**) training on small-diameter containers, and (**b**) training on large-diameter containers. Solid black and dashed line represent the RMSE with standard deviation in shaded region.

In practice, it is likely that a user will encounter a container in the field with dimensions that the ToF-CNN has not been trained on. We investigate the effect of the training/testing dataset (distribution) overlap by first sorting the dataset based on container diameter, and then selecting some fraction of the smallest or largest diameters as a training set $\left\{X_{train},Y_{train}\right\}\subset \left\{X,Y\right\}$ and then selecting the remaining samples to be the test set $\left\{X_{test},Y_{test}\right\}\subset \left\{X,Y\right\}\;:\left\{X_{test},Y_{test}\right\}\cap \left\{X_{train},Y_{train}\right\}=\emptyset$. We then evaluate the prediction performance on the testing set, the training set, and the total dataset, including data used in training and not used in training. Figure 7 shows the RMSE of ToF trained on the smallest diameter containers (Figure 7a), and largest diameter containers (Figure 7b), for the test data (purple), the training data (blue), and the full data, i.e., training + test data (green). It can be observed from Figure 7 that training the model with a small amount of training data produces low training RMSE and much higher test RMSE, suggesting likely overfitting, i.e., that the models are unable to generalize well to unseen data.

**Figure 8:**
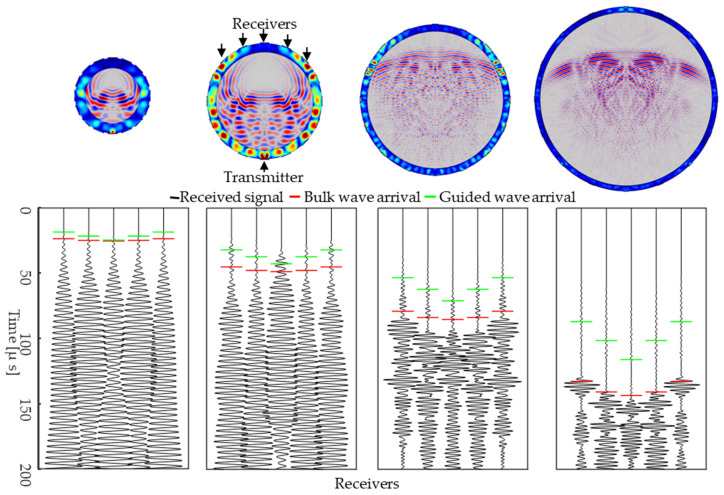
Wavefield snapshot and waveform for different diameter containers, 35 mm, 70 mm, 125 mm, and 212 mm. The bulk wave and first guided wave arrivals are indicated by red and green lines, respectively.

Comparing Figure 7a,b, we observe that training on the smallest containers and testing on the largest containers results in significantly lower test RMSE, compared to training on the largest containers and testing on the smallest containers. Additionally, we observe that, when training on the smallest diameter containers, increasing the training dataset size results in a consistent decrease in the mean test error (Figure 7a). Alternatively, when training on the largest diameter containers, increasing the training dataset size had little effect on the test RMSE until the training set included >80% of the largest containers. To understand why, we observe example wavefield snapshots and waveforms for different diameter containers in Figure 8. We indicate the theoretical bulk wave (red) and first guided wave (green) arrivals. We observe that waveforms from small-diameter containers have similar bulk and guided wave arrival times, making it challenging to distinguish between them. In contrast, for larger containers, bulk and guided waves can be separated comparatively easily. Therefore, for the ranges of dimensions and material properties that we investigate, we contend that small-diameter data has higher complexity compared to large-diameter data. The ToF-CNN is able to learn the complex features from small-diameter data and extrapolate it to large-diameter data, whereas training on large-container data results in lower prediction accuracy for small-diameter data. These results provide important insights into the effect of dataset size and dataset type on predicted ToF error.

### 3.3. Influence of Medium Sound Speed

**Figure 9:**
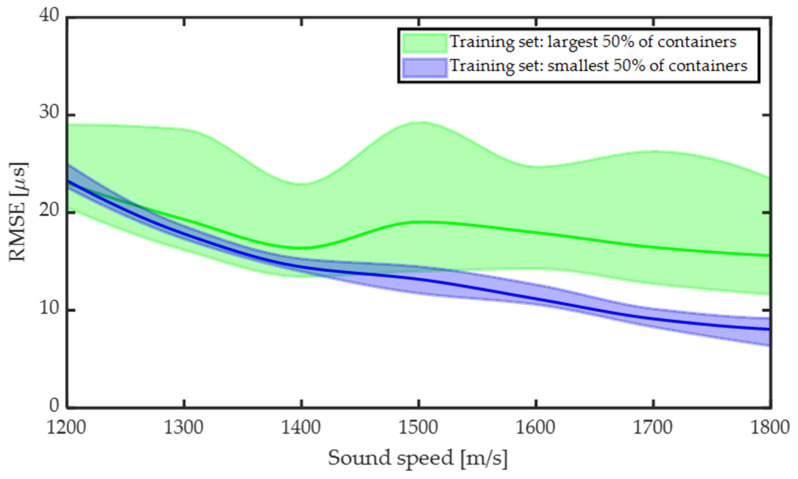
ToF prediction for different speeds when the model is trained on 50% of data for small and large containers. Solid lines represent the RMSE with standard deviation in shaded region.

We have further investigated the viability of the ToF-CNN on containers with different internal material sound speeds. Figure 9 shows the predicted ToF RMSE for different sound speeds of the enclosed material. The training was performed on the smallest 50% and largest 50% diameter data with an acoustic speed of 1498 m/s (water at standard temperature and pressure), and the model was tested on data with sound speed range in the range 1200 m/s to 1800 m/s. Results show that the RMSE decreases monotonically with increasing sound speed for the model trained using a small diameter. This is likely due to the fact that when testing on larger diameters, higher sound speeds will bring the guided and bulk arrival times closer together, which is more similar to the small-diameter data used in training. Alternatively, for the large-diameter data, we observe that the RMSE decreases with increasing sound speed, but not monotonically, and there are large variations in RMSE for all sound speeds. This variation is due to the fact that for many of the containers (especially small), higher sound speed results in similar bulk and guided arrival times. As a result, there is significant interference between the two and it is challenging for the CNN to distinguish them when it has not encountered this in the training data. Thus, these results validate the broader generalizability of the model when training the model on a small-diameter dataset.

### 3.4. Influence of Including Data Augmentation

**Figure 10:**
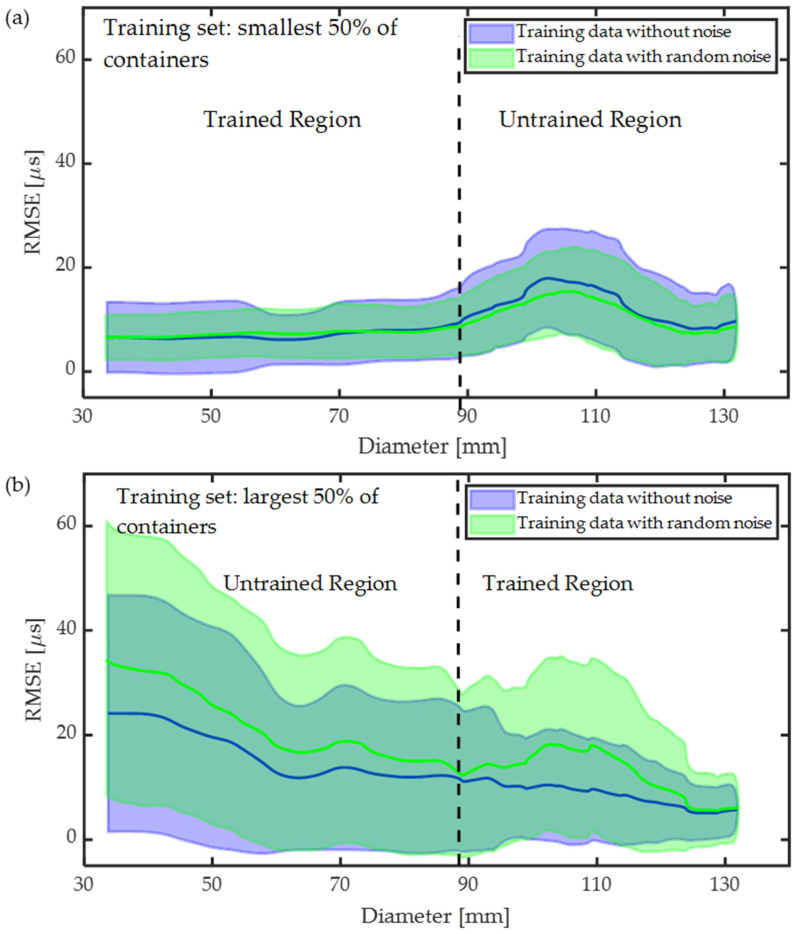
Influence model prediction performance by adding noise: (**a**) training on data (including different velocities) from the smallest 50% of containers, (**b**) training on the largest 50% of containers. Solid lines depict the RMSE with standard deviation in shaded region.

One method for overcoming overfitting is to increase the number of independent training samples. However, this task may not be possible in practice due to the burden of collecting additional experimental data. Alternatively, the training dataset can be augmented or made artificially larger by duplicating and modifying training samples. Here, we test the effect of augmenting the training dataset by duplicating training samples and adding Gaussian noise to the signals. The description of adding noise to the signal is discussed in Section 2.1. An example of the original waveform and noisy waveform is shown in Figure 2. We consider two scenarios: first, the ToF-CNN is trained on data from the smallest 50% of the containers, and second, the ToF-CNN is trained on data from the largest 50% of the containers, as shown in Figure 10a and Figure 10b, respectively. We observe that the model performs better when training on small-diameter data versus training on large-diameter data using augmented samples in the training dataset. Additionally, including noisy data in the training process improves the prediction accuracy for test data when training on small-diameter data (Figure 10a). Interestingly the performance degrades when training on the large-diameter data (Figure 10b). Degradation in the performance of the model trained on large-diameter data is caused by the added noise, which distorts the bulk wave signals, and the ToF-CNN cannot learn features from the training data. Additionally, the model may not be sufficiently complex to represent the relationship between the feature and the outcome. However, the trend follows the same as discussed in Section 3.2.

## 4. Conclusions

We investigate a technique for measuring acoustic bulk wave ToF within closed containers via a ToF-CNN. In closed containers, guided waves propagate through the container walls and interfere with the bulk waves, which makes it challenging to identify the bulk wave. We consider the applicability of a ToF-CNN technique for realistic applications where the test containers encountered in the field may have different dimensions or contain different materials from those used to train the ToF-CNN. The results show low measurement error in test data when enough data is used in training. The impact of training data set size on ToF-CNN is understood, and the influence of the type of container required to train the model is investigated. It was found that training the model with small-diameter data provides higher prediction accuracies when testing on large-diameter data, compared to training on large-diameter data and testing on small-diameter data. It was also observed that the model is robust to noise and speed variation when training the model considering small-diameter data.

To further enhance the data variability in the ToF-CNN model, data augmentation to other variables can be considered, such as transducer positioning. In practice, it is highly possible that the transducers are not positioned equidistantly. Transducer misalignment is straightforward, and it can be augmented in the data by delaying or advancing the time from the signal. Although the model shows excellent prediction accuracy for variable speed medium, it is worth including more data sets with a wide variability in speed for a much broader application range.

These results demonstrate the robustness of the ToF-CNN measurement technique and its limitations in terms of how far the test set parameters can be from those of the training database and still achieve reasonable accuracy. It enables the users to efficiently decide the type of data required to predict ToF within closed containers for various applications such as industrial process control, in situ process monitoring, characterizing phase-change energy storage devices, and material identification.

## Figures and Tables

**Table 1:** Description of the features considered.

Parameter Name	Mean	Standard Deviation
Inner Diameter [mm]	96.278	32.15
Outer Diameter [mm]	108.53	32.49
Thickness [mm]	6.01	1.95
Thin Elastic Layer [mm]	4 × 10^−6^	9.8 × 10^−7^
Sound speed [m/s]	1498	216.02

## Data Availability

The data are not publicly available due to Los Alamos National Laboratory data sharing policy.
